# Remarks on the Milk Sickness, with Cases

**Published:** 1840-12-12

**Authors:** John R. Buck

**Affiliations:** Memphis, Tennessee


### *Remarks on the Milk Sickness, with cases.*

BY JOHN R. BUCK, M. D.

To the Editors of the Medical Examiner.

*Gentlemen,*—In No. 23, Vol. 2, of your journal, I noticed an article on Milk Sickness, and a call on your subscribers to furnish you with an additional account of the disease. Having recently met with some half-dozen cases, my own among the number, in which the symptoms were well marked, I send you the reports of them, If you think them worthy of publication, they are at your service.

*Case 1st.*—Mrs. T———, æt. 30, fat, of lymphatic appearance, the mother of four children, had been slightly indisposed for some days, with sickness of the stomach, slight tenderness, and swelling of the abdomen; these symptoms she referred to the return of the menses, which had taken place a few days before, and were much more copious than usual.

She continued to attend to her household affairs until the afternoon of the 17th October, when violent vomiting came on; she took during the night, at different times, in all, about one and a half grains of morphia, each dose allaying the vomiting for several hours. On the morning of the 18th, the family physician was sent for, a large blister was applied over the epigastric region, and twenty grains of calomel given her; sinapisms to her feet. Her pulse was at this time, as I was told by the attending physician, about one hundred and ten, small and feeble; great tenderness over the whole abdominal region; slight cephalalgia. The calomel was repeated in the afternoon, injections were administered, but nothing could overcome the obstinate constipation. The vomiting continued throughout the day, and the next night with but little intermission, notwithstanding opium was given in doses of four or five grains. I was requested on the morning of the 19th, by her attending physician, to see her; she was then lying upon her back, a general subsidence of the features; not such as attends the removal of disease, but a sinking of the eyes and cheeks, and still an anxiety in her looks which is always indicative of great danger. Pulse too feeble to be counted, respiration twenty-six in the minute; extremities cold, covered with a clammy sweat; a peculiar fœtor of the breath. Stimulants were used both externally and internally, mustard foot bath, frictions with dry mustard; brandy toddy and other stimulants were used internally. She sank gradually from this time until her death, which took place at 2 o'clock the next morning. She was comatose for several hours previous to her death. No examination was made.

*Case 2d.*—Mrs. R———, æt. 28, sister-in-law to Mrs. T., who had been living with and attending her illness, was attacked in precisely the same manner on the afternoon of the 21st. I was requested to see her, and found her vomiting; skin warm; pulse one hundred and twenty, moderately full; some tenderness over the whole abdominal region; slight cephalalgia; a general feeling of malaise. I gave her ten grains of calomel and one of opium, had a mustard plaster put over the stomach, and left four pills of morphia to be given during the night if the vomiting was not checked.

At my visit next day, at 5 P. M., I found that she had spent a very bad night. She had taken all the pills without any relief. Twenty grains of calomel had been given the next morning, by the family physician. Two injections had also been given, but without producing any operation from the bowels. Her condition was decidedly worse than it had been the day before. The same change of countenance, the same peculiar fœtor of the breath that occurred in case 1st, was now present. Skin cool, slightly moist; pulse one hundred and thirty, small and feeble; headach increased; tenderness over the abdominal region greatly increased; the weight of the bed cloths painful.

*Practice.*—Sinapisms to the calves of the legs, seidlitz powders. She slept about half an hour while the mustard was drawing. She was cupped during the night, which relieved her for a few hours; it was repeated, but not with the like good effect. The calomel had produced its effects upon the salivary glands. On my visit to her the next day, she complained of a burning sensation in the stomach.

Bicarbonate of soda ℥ss. was given, which afforded her more relief than any thing she had taken since her attack. She slept soundly after it for an hour and a half; awoke with some retching, but no vomiting; the soda was repeated with the same good effects. This treatment was continued during the night; the vomiting was entirely relieved by it. She complained the next morning of great prostration. She still had sickness at the stomach, with occasional retching, but it seemed to be owing more to the effects of salivation than to the primary disease. A small dose of laudanum and camphor was administered, and afterwards, during the day, small doses of spirits of camphor; this treatment produced a most happy effect. Some purgative pills, composed of rhubarb, aloes, and soap were given, which operated finely upon the bowels; her appetite returned, and her convalescence was rapid. I saw her a few days since, and though she looks entirely well, she says that she is yet occasionally troubled with sick stomach.

*Case 3d.*—Indy, a coloured girl, æt. 14, unmarried, had been for several weeks living in the same house with case 1st and 2d, and had waited upon Mrs. T. the first day of her sickness, On the evening of the 19th she first complained of her head and stomach. I did not see her until several days subsequent to her attack. She had then taken several doses of calomel; soda powders with only half the acid; sinapisms had also been applied over the region of the stomach and to the ankles. I found her complaining of a most violent pain in the abdominal region; constant disposition to vomit; pulse one hundred and ten, and full. I suggested to the attending physician the propriety of cupping her. This was tried, and it had the happiest effect. The operation was performed in the early part of the evening; she slept well all night, and continued to improve for several days, when I was again sent for to see her. She had had a return of the vomiting. I gave her a tea spoonful of the spirits of camphor, which quieted her stomach, and left a dose of purgative pills to be taken. The pills operated well, and she soon recovered.

*Case 4th.*—Of the history of this case I know but very little. It has never been strictly under medical treatment, and of course no accurate history can be obtained. Mr. T., æt. 34, consort of case 1st, was attacked some time in September last with nausea and vomiting; he has scarcely been free from it a day, and often not one hour in the day, since. Naturally a stout, healthy, muscular man, he has dwindled almost to a skeleton. In his previous attacks, for this is not the first, he has generally succeeded in checking the disease by cold affusions, drinking lime water, active purgatives, &c., but they have all been tried in this attack without doing any good. Active exercise of any kind will bring on an attack of vomiting. So long as he remains perfectly quiet in a recumbent position he feels comfortable. It should be mentioned, however, that at no period since he was first attacked has anorexia been complete, and though he has abstained from the use of milk, butter and beef have both been freely used by him. Several other members of the same family have had slight attacks of the same disease during the present fall, but they have yielded very readily, so soon as the bowels have been freely opened. Mr. T. informed me, that about a year since he lost a fine healthy negro woman with the same disease.

*Case 5th.*—In giving the history of this my own case, I shall be more particular both as to the symptoms and treatment than I have been in the preceding cases. I had felt dull and heavy for several days, with diminution of appetite. I awoke on the morning of the 10th with slight nausea, weakness of the limbs, slight tremours upon getting out of bed. I remained in my room, but not in bed, during the day, took very little nourishment, no medicine; I drank about bed time pretty freely of brandy toddy, but without relieving the nausea. I slept pretty well that night, but awoke the next morning with the most distressing nausea; slight cephalalgia; pulse 85, moderately full; throbbing of the abdominal arteries; respiration natural; skin natural; bowels constipated; no thirst; no appetite; I remained in this condition without taking any thing until 2 o'clock, when I took a large dose of sulphate of magnesia. This operated some four or five times, but did not relieve the nausea. At bed-time I took a dose of ipecacuanha, which operated almost immediately, and continued with scarcely any intermission until the next morning. So violent and constant had been the vomiting during the night, that I scarcely felt able to turn myself in bed; pulse pregnant and feeble. I attempted to take a solution of the *carb. sodæ*, but it vomited me before I could drink the half of it. I then took a soda powder with only half of the acid, leaving half the alkali free; this, drank in a state of effervescence, is not unpleasant, and relieves the sickness at the stomach more effectually than any thing else that I have ever tried; the relief is temporary, and it requires to be frequently repeated. About an hour after each dose, a burning sensation in the stomach would commence, and with it nausea, sometimes vomiting, which would continue until the powder was repeated, always omitting half the acid. This treatment was continued, (no nourishment taken in the meantime,) until the evening of the 15th. My bowels then had not been opened since the 11th. The nausea had been almost entirely relieved; some little appetite. I took a large dose of the cream of tartar, which opened my bowels freely; an occasional soda powder for two days longer ended the treatment. I have met with two other cases during the present season. The farm upon which the four first cases occurred is situated in Wolf River Bottom, about one mile above its entrance into the Mississippi. The land is heavily timbered, and covered with a dense undergrowth of native vegetation. The fatality to stock ranging in this particular neighbourhood is very great. The poison seems to remain latent in the system until developed by excess of some kind, either in eating, drinking, or active bodily exercise. In cattle, active exercise, so that they become heated, is necessary to the development of the disease. Two dogs, after eating the flesh of an animal dying of it, sickened and died in twenty-four hours. The particular vegetable upon which this disease depends, has never yet been discovered; indeed, it is even a mooted point, upon which I shall not pretend to decide, whether the poison be a vegetable or a mineral. Many facts have been brought forward in support of either opinion; but, so far, the greatest number of facts seems to be in favour of its vegetable origin.

It is said by some writers on the subject to have been produced by drinking water. Admitting this to be the fact, may not the water be impregnated with decayed vegetable matter? for if the disease depended upon a mineral impregnation of the water, would it not be increased instead of being totally eradicated, as is certainly the case, by cultivation? Another argument used by a writer advocating the mineral origin of this disease, is, that, after being once checked, it is liable to return without any additional quantity of the poison being taken into the system, and goes on to say that this is not the case with any vegetable poison. Now, with all due deference to the gentleman, I must beg leave to differ with him in the latter opinion. Every physician, whose practice extends to the country, has doubtless seen cases proving the contrary. The effects of poison from the common poison oak, may be, to all appearances, removed, and re-appear days and even weeks after, upon the face, hands, or some other part of the body. Marsh miasm produces intermittent fever. This may be to all appearances cured, and the patient removed from the infected district, and yet the disease may and often does return. Will it be concluded from this fact that marsh miasm is a mineral, and not a vegetable poison? The soil upon which the above mentioned cases occurred is entirely alluvial, and consequently composed almost wholly of decayed vegetable matter. I am informed by an intelligent medical friend, Dr. Walker, that a few miles below Memphis, in the immediate vicinity of the embryo city of Fort Pickering, the ground is fairly bleached with the bones of cattle. The soil and the growth are the same as that already described. Dr. W. has met with but one case during the present season; and upon inquiry of the woman with whom the patient boarded, was told that the milk of which the man had been in the habit of drinking was much thinner than ordinary, and that the calf, after sucking freely, would vomit like a child.

*Memphis*, (*Tennessee*,) 30th Nov., 1840.

